# RAB39B is redistributed in dementia with Lewy bodies and is sequestered within aβ plaques and Lewy bodies

**DOI:** 10.1111/bpa.12890

**Published:** 2020-08-25

**Authors:** David J. Koss, Odeta Bondarevaite, Sara Adams, Marta Leite, Flaviano Giorgini, Johannes Attems, Tiago F. Outeiro

**Affiliations:** ^1^ Translational and Clinical Research Institute Faculty of Medical Sciences Newcastle University Framlington Place Newcastle Upon Tyne NE2 4HH UK; ^2^ Department of Genetics and Genome Biology University of Leicester University Road Leicester LE1 7RH UK; ^3^ Department of Experimental Neurodegeneration Center for Biostructural Imaging of Neurodegeneration University Medical Center Goettingen Goettingen Germany; ^4^ Max Planck Institute for Experimental Medicine Goettingen Germany

**Keywords:** alpha‐Synuclein, Lewy body dementia, Parkinson’s disease, proteostasis, RAB39B

## Abstract

Loss of function mutations within the vesicular trafficking protein Ras analogy in brain 39B (RAB39B) are associated with rare X‐linked Parkinson’s disease (PD). Physiologically, RAB39B is localized to Golgi vesicles and recycling endosomes and is required for glutamatergic receptor maturation but also for alpha‐Synuclein (aSyn) homeostasis and the inhibition of its aggregation. Despite evidence linking RAB39B to neurodegeneration, the involvement of the protein in idiopathic neurodegenerative diseases remains undetermined. Here, analysis of the spatial distribution and expression of RAB39B was conducted in post‐mortem human brain tissue from cases of dementia with Lewy bodies (DLB, n = 10), Alzheimer’s disease (AD, n = 12) and controls (n = 12). Assessment of cortical RAB39B immunoreactivity using tissue microarrays revealed an overall reduction in the area of RAB39B positive gray matter in DLB cases when compared to controls and AD cases. Strikingly, RAB39B co‐localized with beta‐amyloid (Aβ) plaques in all cases examined and was additionally present in a subpopulation of Lewy bodies (LBs) in DLB. Biochemical measures of total RAB39B levels within the temporal cortex were unchanged between DLB, AD and controls. However, upon subcellular fractionation, a reduction of RAB39B in the cytoplasmic pool was found in DLB cases, alongside an increase of phosphorylated aSyn and Aβ in whole tissue lysates. The reduction of cytoplasmic RAB39B is consistent with an impaired reserve capacity for RAB39B‐associated functions, which in turn may facilitate LB aggregation and synaptic impairment. Collectively, our data support the involvement of RAB39B in the pathogenesis of DLB and the co‐aggregation of RAB39B with Aβ in plaques suggests that age‐associated cerebral Aβ pathology may be contributory to the loss of RAB39B. Thus RAB39B, its associated functional pathways and its entrapment in aggregates may be considered as future targets for therapeutic interventions to impede the overall pathological burden and cellular dysfunction in Lewy body diseases.

## Introduction

Dysfunctions in protein homeostasis (proteostasis) play a role in the pathogenesis of neurodegenerative diseases including Parkinson’s disease (PD), Dementia with Lewy bodies (DLB) and Alzheimer’s disease (AD). Besides protein aggregation, alterations in protein trafficking and subcellular distribution are common cellular pathologies in these and other neurodegenerative disorders. Many of the mechanisms which control cellular trafficking pathways and their key regulators are altered as a consequence of disease progression (1, 2).

Vesicular trafficking is essential for the maturation, recycling and degradation of proteins, as well as for the delivery of lipids to specific membranes (15). The endosomal pathways utilized for trafficking are tightly regulated by the Ras analogy in brain (RAB) GTPases superfamily. RAB proteins cycle between cytoplasmic and membrane bound states, and once bound, interact with numerous effectors such as coat proteins, molecular motors, structural tethers and docking/fusion proteins (29). Accordingly, the profile of RAB proteins associated with a particular vesicle defines a specific functional identity and thus ensures that correct processing of cargo ensues. Consequently, RAB protein dysfunction can be anticipated to have widespread consequences as the misdirection of trafficked cargo or the loss of vesicular function may impact upon virtually all cellular processes.

Indeed, many RAB proteins are associated with the progressive pathology of neurodegenerative diseases, although few have been demonstrated as pathogenic in their dysfunction (15). Of these, loss of function mutations in the *RAB39B* gene are associated with early onset PD (5, 16, 19, 28, 35). Post‐mortem examination of these cases reveal typical subcortical LBs but also neurofibrillary tangles (NFTs) alongside more widespread cortical LB deposition (35).

Although not fully resolved, several studies have begun to establish the physiological functions of RAB39B. RAB39B is predominantly expressed in neuronal cells and is localized to the endoplasmic reticulum, Golgi and trans‐Golgi recycling endosomes and RAB39B‐positive vesicles extend along the neuritic processes into synaptic compartments (10, 13, 18). Functionally, RAB39B is required for alpha‐amino‐3‐hydroxy‐5‐methyl‐4‐isoxazole propionic acid receptor (AMPAR) subunit GluR maturation (22) and has been implicated in the aggregation and toxicity of aSyn (14, 35). Furthermore, via its interaction with the chromosome 9 open reading frame 72 (C9orf72) gene product (6, 9, 26), implicated in amyotrophic lateral sclerosis (ALS) and frontotemporal dementia (FTD), additional roles for RAB39B in postsynaptic GluR subunit expression (37) and autophagy (26) have also been established.

A reduction in the levels of RAB39B in the substantia nigra and prefrontal cortex has been recently reported in idiopathic PD cases, further confirming its association with aSyn‐mediated neurodegeneration (11). However, despite the latter and the genetic linkage of RAB39B in a rare, familial PD variant, RAB39B expression in DLB and AD has not been investigated.

Hence, we quantified RAB39B expression in post‐mortem human tissue obtained from clinically and neuropathologically confirmed idiopathic cases of AD and DLB, as well as aged‐matched non‐diseased controls. We found an overall reduction in the area of RAB39B expression in the cortex alongside a depletion of the cytoplasmic pool of RAB39B in DLB cases. Additionally, RAB39B co‐localized with LBs and Aβ plaques highlighting the proteins sequestration as part of the occurrence of disease‐associated neuropathology. Collectively, our study supports the involvement of RAB39B dysregulation in the pathogenesis of LBDs, where the progressive loss of functionality as a consequence of its inclusion in aggregates leads to the increased vulnerability for further protein deposition, disease onset and progression.

## Materials and Methods

### Human post‐mortem brain tissue

A study cohort of post‐mortem human brains comprising of clinico‐pathologically classified AD (n = 12), DLB (n = 10) and non‐neurodegenerative control cases (Con, n = 12) was obtained from the Newcastle Brain Tissue Resource (NBTR). For histology and tissue micro‐array (TMA), tissue sections were prepared from the right hemisphere of the brain fixed for 4–6 weeks in 4% paraformaldehyde. Corresponding frozen temporal cortex tissue (Brodmann’s area (BA) 21/22) was obtained from the left hemisphere, dissected in a coronal plane and snap frozen between copper plates at −120°C prior to being stored at −80°C. As a consequence of limitations in tissue availability, it was not possible to obtain both fixed and frozen tissue for all cases (see Table 1 and S1 for full details). Comparative analysis of age and post‐mortem interval (PMI) between disease groups determined no significant difference in either measure (*P *> 0.05).

**Table 1:** Human tissue cohort. Human cases used in tissue microarray and immuno‐blot separated by disease classification according to non‐diseased controls (Con), Alzheimer’s disease (AD) and dementia with Lewy bodies (DLB). Case numbers (n), sex, age, post‐mortem interval (PMI), neurofibrillary tangle (NFT) Braak stage, Thal phase, Consortium to Establish a Registry for Alzheimer’s disease (CERAD), the National Institute of Aging––Alzheimer’s Association (NIA‐AA) criteria, Lewy body (LB) Braak stage and McKeith criteria are provided. For age and PMI both range and mean ± SEM are provided. For numerical scores of pathology, range and percentage composition are given. For McKeith criteria, only percentage composition is given, where cases free of LBs (No LB), amygdala predominate (Amyg), limbic predominate (Limb) and neocortical predominate (Neo) are indicated

Disease	N	Sex (% male)	Age (years)	PMI (hours)	NFT Braak stage	Thal phase	CERAD	NIA‐AA	LB Braak stage	McKeith criteria
*Tissue microarray*										
Con	12	50%	52‐99	5‐102	0‐III	0‐4	0	0‐1	0‐2	100%‐No LB
			78.4 ± 3.6	48.25 ± 8.8	33.3%‐0	50%‐0	100%‐0	50 %‐ 0	91.6%‐0	
					8.4%‐ I	25%‐1		50 %‐1	8.4%‐1	
					33.3%‐II	8.4%‐2				
					25%‐III	8.4%‐3				
						8.4%‐4				
										
AD	10	50%	77‐92	5‐72	VI	4‐5	3	3	0	80%‐No LB
			83.8 ± 1.7	40.2 ± 7.1	100%‐VI	10%‐4	100%‐3	100%‐3	100%‐0	20%‐Amyg
						90%‐5				
										
DLB	8	75%	71‐91	8‐84	II‐VI	*3‐5	0‐3	*1‐2	*5‐6	12.5%‐ Limb
			79.4 ± 2.2	44.9 ± 9.9	25%‐II	40%‐3	37.5%‐0	50%‐1	16.7%‐5	87.5%‐Neo
					37.5%‐III	40%‐ 4	12.5%‐1	50%‐ 2	83.3%‐‐6	
					12.5%‐IV	20%‐ 5	25%‐2			
					12.4%‐V		25%‐3			
					12.5%‐VI					
*Immuno‐blot*										
Con	10	59.3%	52‐99	5‐102	0‐III	0‐4	0	0‐1	0‐2	100%‐No LB
			77.3 ± 4.3	53.2 ± 9.9	40%‐0	50%‐0	100%‐0	50%‐0	90%‐0	
					10%‐I	20%‐1		50%‐1	10%‐2	
					30%‐II	10%‐2				
					30%‐III	10%‐3				
						10%‐4				
										
AD	12	59.3%	77‐93	5‐74	VI	4‐5	3	3	0	75%‐No LB
			84.7 ± 1.6	36.1 ± 6.5	VI‐100%	8.3%‐4	100%‐3	100%‐3	100%‐0	25%‐Amyg
						91.7%‐5				
										
DLB	10	75%	71‐91	8‐99	II‐VI	*1‐5	0‐3	*0‐2	*5‐6	10%‐Limb
			78.1 ± 2	50.5 ± 9.5	20%‐II	14.2%‐1	50%‐0	12.5%‐0	12.5%‐5	90%‐Neo
					50%‐III	28.6%‐3	10%‐1	50%‐ 1	87.5%‐‐6	
					10%‐IV	42.9%‐4	20%‐2	37.5%‐2		
					10%‐V	14.2%‐5	20%‐3			
					10%‐VI					

* Composition based on available data.

The final clinico‐pathological diagnoses were established by combining clinical data and neuropathological diagnoses, which were based on assessment of brain tissue according to the National Institute of Aging––Alzheimer’s Association (NIA‐AA) criteria (24), including Braak NFT staging (3), Thal phases (32) and Consortium to Establish a Registry for Alzheimer’s Disease (CERAD) scoring (23), as well as Braak LB stages (4) and Newcastle/McKeith Criteria (20, 21) (Tables 1 and S1). The use of human tissue throughout this study was in accordance with Newcastle University Ethics Board (The Joint Ethics Committee of Newcastle and North Tyneside Health Authority, reference: 08/H0906/136).

### Tissue microarrays (TMAs)

High‐throughput regional quantification of the spatial expression of RAB39B within the brain was established via an in house developed TMA, as described previously (34). Briefly, donor paraffin embedded tissue blocks were sampled via 3 mm cylindrical tissue cores taken from: pre‐frontal cortex (pFc‐ BA 9, 10/46), frontal cortex (Fc‐ BA 8,9), cingulate cortex (Cc‐ BA 24, 32), caudate, putamen, globus pallidus, amygdala, insular cortex (IC‐ BA 13), motor cortex (Mc‐ BA4), thalamus, entorhinal cortex (Ec‐ BA28), temporal cortex (Tc‐ BA 21, 22, 41/42), parietal cortex (Pc‐ BA 22, 40), occipital cortex (Oc‐ BA 17, 18, 19, 37) and white matter of the pFc, Tc, Pc and Oc in order to produce paraffin‐embedded TMA blocks for each case. TMA blocks contained 40 individual tissue punches, representing nine cortical and six subcortical regions, from which 6 µm thick sections were cut and mounted on glass slides for subsequent immunostaining.

Slide mounted sections were stained within three batch staining runs, each comprising of approximately one third of the cases from each neurodegenerative disease category (Con, AD and DLB). TMA slides were baked at 60°C for 1 hour prior to being dewaxed in xylene, rehydrated in descending concentration of ethanol (5 minutes immersion) and washed in Tris‐buffered saline (TBS; 5 mM Tris, 145mM NaCl and pH 7.4). Slides were treated with microwave‐assisted antigen retrieval (800 W, 10 minutes) in citrate buffer (10 mM Citric acid, 0.05 % Tween 20 and pH 6) before endogenous peroxidases were quenched in H_2_O_2_ (3%, 20 minutes submersion). Following consecutive washes in TBS and TBS containing Tween‐20 (TBST: 5 mM Tris, 145 mM NaCl and 0.01% Tween‐20 pH 7.4), slides were incubated with a rabbit anti‐RAB39B polyclonal antibody (1:100, 12162‐AP, Proteintech, Manchester, UK) in TBS for 1 hour. Visualization of immunoreactivity was achieved via the MENAPATH HRP polymer detection kit (Menarini diagnostics, Wokingham, UK) and 3,3′‐ Diaminobenzidine (DAB) chromogen with appropriate TBS and TBST washes was performed between steps. Slides were co‐stained with hematoxylin prior to being dehydrated in ethanol, cleared in xylene and mounted in dibutylphthalate polystyrene xylene (DPX).

### TMA quantification

TMA slides labeled for RAB39B were subject to a semi‐automated microscope‐based capture system (Nikon Eclipse 90i microscope, DsFi1 camera and NIS elements software V 3.0, Nikon) and image analysis protocol (as previously detailed (34)). By means of a motorized software controlled stage, each tissue punch was assessed via multiple images captured at x100 magnification, forming a 3 x 3 image grid, with 15% overlap between adjacent fields such that the total area sampled for each punch was 1.7 mm^2^. Following the capture of all 40 punches, image grids were inspected and regions of interests (ROIs) applied to exclude overt tissue abnormalities (folds and tears), tissue edges or erroneously included white matter regions. Any punch which was too damaged or missing from the array was noted and factored into the analysis. Within the established ROIs, a single restriction threshold based on RGB values was applied to capture immunoreactivity generating a binary signal image from which percentage area coverage could be calculated. For areas represented by more than one tissue punch, the resulting measures of area coverage were pooled and the mean for each region calculated.

### Immunofluorescence histochemistry

Additional TMA slides were co‐labeled for either phosphorylated tau (AT‐8), beta amyloid (4G8) or aSyn (Syn‐1) pathology and RAB39B (Table 2 for staining details and S1 for the specific cases used). For immunofluorescent staining, slides were dewaxed and cleared as above, before treated appropriately for optimal antigen retrieval (see Table 2), blocked in 5% normal goat serum (NGS) containing TBS (1 hr, at RT), incubated in primary antibody containing 5% NGS TBS (overnight, 4°C) and subsequently in secondary antibody (1:500 for both goat anti‐mouse IgG Alexa 488 and goat anti‐rabbit IgG Alexa 594 in 5% NGS TBS for 1 hr, at RT). Upon completion of antibody staining, slides were treated with Sudan black (5 minutes, immersion) to quench autofluorescence and Prolong Diamond mounting media containing DAPI (Fisher Scientific, Loughborough, UK) added before being coverslipped. Fluorescently labeled slides were imaged via a wide‐field fluorescence microscope system (Nikon Eclipse 90i microscope, DsQi1Mc camera and NIS elements software V 3.0, Nikon) or via confocal microscope (Lecia SP 8, LAS X software, Leica‐microsystems).

**Table 2:** Pathology antigen retrieval and antibody table. Optimal conditions for the detection of hyperphosphorylated tau, beta amyloid and alpha Synuclein are provided alongside antibodies used for their detection, the corresponding dilution of each antibody and their supplier. Phosphorylated (p) serine (Ser) and Threonine (Thr) residues are provided for AT8 and amino acid (aa) epitope provided for 4G8 and Syn‐1

Pathology co‐stain	Antigen‐retrieval	Pathology antibody	Dilution	Supplier
Hyperphosphorylated Tau	Citrate buffer (10 minutes microwave heated)	AT8 (pSer202/pThr205)	1:4000	Innogenetics, Ghent, Belgium
Beta Amyloid	Citrate buffer + 90% Formic acid (1 hour submersion)	4G8 (aa18‐aa22)	1:15 000	Biolegend, London, UK
Alpha Synuclein	Citrate buffer + EDTA buffer (10 mM EDTA, pH 8, 2 minutes pressured heating)	Syn‐1 (aa91‐99)	1:1000	BD Biosciences, San Jose, CA, USA

### Tissue homogenization and fractionation

Crude temporal cortex lysates (~100 mg) were generated from frozen cortical tissues blocks homogenized (1:10, W:V) in ice‐cold Triethylammonium bicarbonate buffer (TEAB, pH 8.5, Sigma, Gillingham, UK) containing complete protease inhibitor cocktail and phostop tablets (each at 1/10ml, Sigma), prior to being stored at −80°C.

For subcellular fractionation, aliquots (100 µL) of crude TEAB lysates were centrifuged (16 000 rcf, 40 minutes, 4°C), supernatant collected and stored as soluble cytoplasmic fraction. The resulting pellets were twice washed in excess (500 µL) Tris–HCl buffer (50 mM Tris–HCl, 150 mM NaCl and 2 mM EDTA, pH 7.4) and centrifuged (16 000 rcf, 40 minutes, 4°C). Washed pellets were respun and membrane bound components extracted in 5% SDS containing Tris–HCl buffer (pH 7.4), cleared via centrifugation (16 000 rcf, 40 minutes, 10°C) and supernatant stored as membrane soluble fraction. Following a twice repeated wash in 5% SDS containing Tris–HCl buffer, insoluble proteins were subsequently extracted in 8M Urea 5% SDS containing Tris–HCl Buffer (pH 7.4), centrifuged and supernatant collected as insoluble fraction. All fractions were stored at −80°C, prior to use.

### Immunoblotting

Immunoblots of crude and subcellular fractions were conducted for the quantification of RAB39B expression. Protein extracts were adjusted to 5 µg/lane for crude and 2 µg/lane for soluble and membrane fractions as per Bradford assay (Sigma) with lithium dodecyl sulphate loading buffer (LDS buffer, Fisher Scientific), reducing agent (Fisher Scientific) and dH_2_O. As a consequence of the interference of urea and SDS within the insoluble fraction with the Bradford assay a set volume of 5 µL/lane was used. All samples were heated at 70°C for 10 minutes prior to being loaded onto 4–12% Bis‐Tris SDS page gels (Fisher Scientific), separated (35 minutes, 200 V constant) in MES buffer (Fisher Scientific) and transferred to nitrocellulose membranes via IBlot (7 minutes, 20 mV; Fisher Scientific). For dot‐blot quantification of aSyn and Aβ, crude lysates were adjusted to 10 µg/dot with dH_2_O and dotted onto 0.45 µm nitrocellulose membranes (GE Healthcare Amersham, Fisher Scientific). All membranes were blocked in 5% milk powder containing TBST for 1 hour at room temperature (RT) and incubated in primary antibody solution (5% BSA containing TBST with rabbit anti‐RAB39B polyclonal antibody or rabbit anti‐phospho‐S129‐aSyn [EP1536Y, ab51253, abcam] or mouse anti‐Aβ [MOAB‐2, M‐1586‐100, 2BScientific, Stonesfield, UK], all at 1:1000, supplemented with 0.05% Sodium Azide) overnight at 4°C and incubated with secondary antibody (1:5000, goat anti‐rabbit or goat anti‐mouse IgG HRP antibody in 5% milk powder containing TBST for 1 hour at RT). Adequate washing in TBST was performed between each step. Immunoreactivity was visualized via enhanced chemiluminescence (1.25 mM Luminol, 30 μM coumaric acid and 0.015% H_2_O_2_), captured with a Fuji LAS 4000 with imaging software (Fuji LAS Image, Raytek, Sheffield, UK) and images saved in 16 bit for analysis and 8 bit for illustration.

Protein loading was determined either by probing membranes with antibodies directed to housekeeping proteins β‐tubulin (1:10 000, ab6046, Abcam) or glyceraldehyde 3‐phosphate dehydrogenase (GAPDH; 1:5000, 14C10, Cell signaling) or by staining for total protein via ponceau S (0.1% ponceau S in 5% acetic acid), where appropriate. Samples were run in batches, such that each blot contained ≥ 3 cases of each neurodegenerative group and thus densitometric blot analysis via ImageJ (NIH) could be adjusted to similar measures of loading controls and normalized within blots to control cases prior to pooling measures between blots.

Additionally, the detection of aSyn (phospho‐S129‐aSyn and Syn‐1, both at 1:1000) and Aβ (4G8; 1:1000) species within the generated subcellular fractions was conducted to further establish proper cellular fractionation and co‐localize RAB39B with relevant pathological aSyn and Aβ species.

### Statistical analyses

All data were subject to Shapiro–Wilk test for the determination of normal or nonnormal distribution and Grubbs’ test for outliers. For TMA analysis, regional cortical comparisons between disease categories were established via two‐Way ANOVA with Bonferroni posttest. For immunoblot analysis, loading control adjusted RAB39B measures were normalized to control cases within blots, pooled between blots and subject to Kruskal–Wallis test of significance and appropriate post hoc comparisons. Associations of experimental measures with age and PMI were conducted via Spearman’s correlation. For all analysis, *P* < 0.05 was assumed as significant.

## Results

### Immunohistochemical detection and quantification of RAB39B

**Figure 1:**
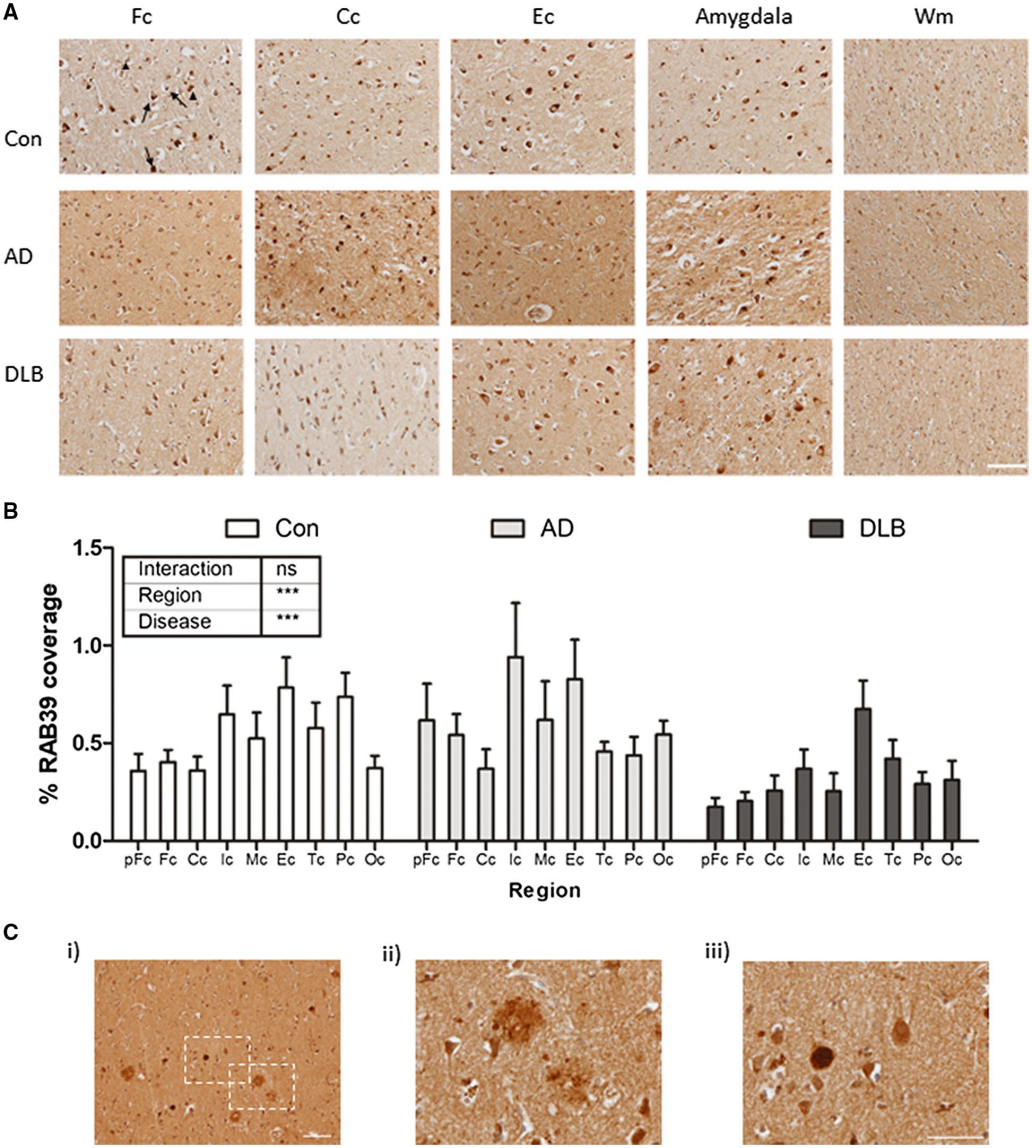
Cortical distribution of RAB39B in the human brain. **A.** Representative 100x images of frontal (Fc), cingulate (Cc) and entorhinal (Ec) cortex from control (Con), Alzheimer’s disease (AD) and dementia with Lewy bodies (DLB) cases. Additional images from the amygdala and frontal white matter (Wm) are also shown. In cortical regions, robust somatic cytoplasmic staining was apparent (arrows) and in some cells RAB39B appeared within intracellular cytoplasmic bodies (arrowhead). **B.** Comparative quantification in Con, AD and DLB cases of the percentage area coverage of RAB39B immunoreactivity in the pre‐frontal (pFc), frontal (Fc), cingulate (Cc), insular (Ic), motor (Mc), entorhinal (Ec), temporal (Tc), parietal (Pc) and occipital (Oc) cortices. Data shown as mean ± SEM and output from two‐way ANOVA is stated, ***P < 0.001 and ****P < 0.0001. **C.** Examples of RAB39B positive neuropathological aggregates in the cortex of one individual DLB case. At 100x magnification, both Aβ plaques (magnified at 400X in ii) and Lewy bodies (LBs; magnified at 400x in iii) were evident. Scale bar in **A** and **C** i = 50 µm and in **C** iii = 100 µm

Immunohistochemical detection of RAB39B in post‐mortem human brain tissue demonstrated a predominantly somatic cytoplasmic cellular localization, with modest staining also present within the neuropil. RAB39B immunoreactivity was observed in large cortical cells, exhibiting a neuronal morphology (Figure 1A, arrow). In a subset of RAB39B positive cells, the staining pattern suggested the presence of RAB39B in intracellular cytoplasmic bodies (Figure 1A, arrowhead). Most cells with comparatively smaller nuclei, suggestive of a glial lineage, appeared to lack robust RAB39B staining. The staining pattern observed was consistent throughout the cortex across all regions examined as well as in the amygdala (Figure 1A and see, Figure S1 for representative images for all regions examined). The preferential expression of RAB39B in neurons and not in glial cells was further supported by the absence of RAB39B positive cell bodies within the white matter areas examined (Figure 1A, see, frontal white matter for example) and is consistent with the expression profile established in mouse brain (12, 13).

Comparison across different groups revealed no overt changes in the cellular distribution of RAB39B, with immunoreactivity remaining consistent between Con, AD and DLB cases. TMA analysis of the RAB39B coverage showed both a significant difference of RAB39B signal between cortical regions (two‐way ANOVA, F_(8,238)_=3.55, *P* < 0.001) and diseases (F_(2,238)_=9.54, *P* < 0.001), although no differences were observed upon individual regional comparisons (Figure 1B). The difference of RAB39B between diseases was largely dependent on the DLB group, as when analysis was limited to the Con and DLB cases only, the statistical outcome remained (regional; *F*_(8,162)_ = 3.68, *P* < 0.001 and disease *F*_(1,162)_ = 14.93, *P* < 0.001), with post hoc analysis reporting a reduction in RAB39B coverage in DLB between controls within the Pc (*P* < 0.05). Thus, the data shows an overall reduction in RAB39B coverage within the cortical regions of DLB cases. Similar comparisons within the subcortical amygdala found no significant difference between diseases (data not shown). No correlations with regional RAB39B coverage and age or PMI were observed (*P *> 0.05). In one DLB case, which was excluded from the TMA analysis, robust RAB39B labeling was detected within abnormal structures consistent with extracellular Aβ plaques (Figure 1C i,ii) and intracellular LBs (Figure 1C i,iii).

### Accumulation of RAB39B within protein aggregates

As indicated by DAB‐based immunohistochemistry, the accumulation of RAB39B within AD‐associated Aβ plaques and LBs associated with LBDs was confirmed via immunofluorescent co‐staining following optimal antigen retrieval methods for plaques, NFTs and LBs (see Table 2).

**Figure 2:**
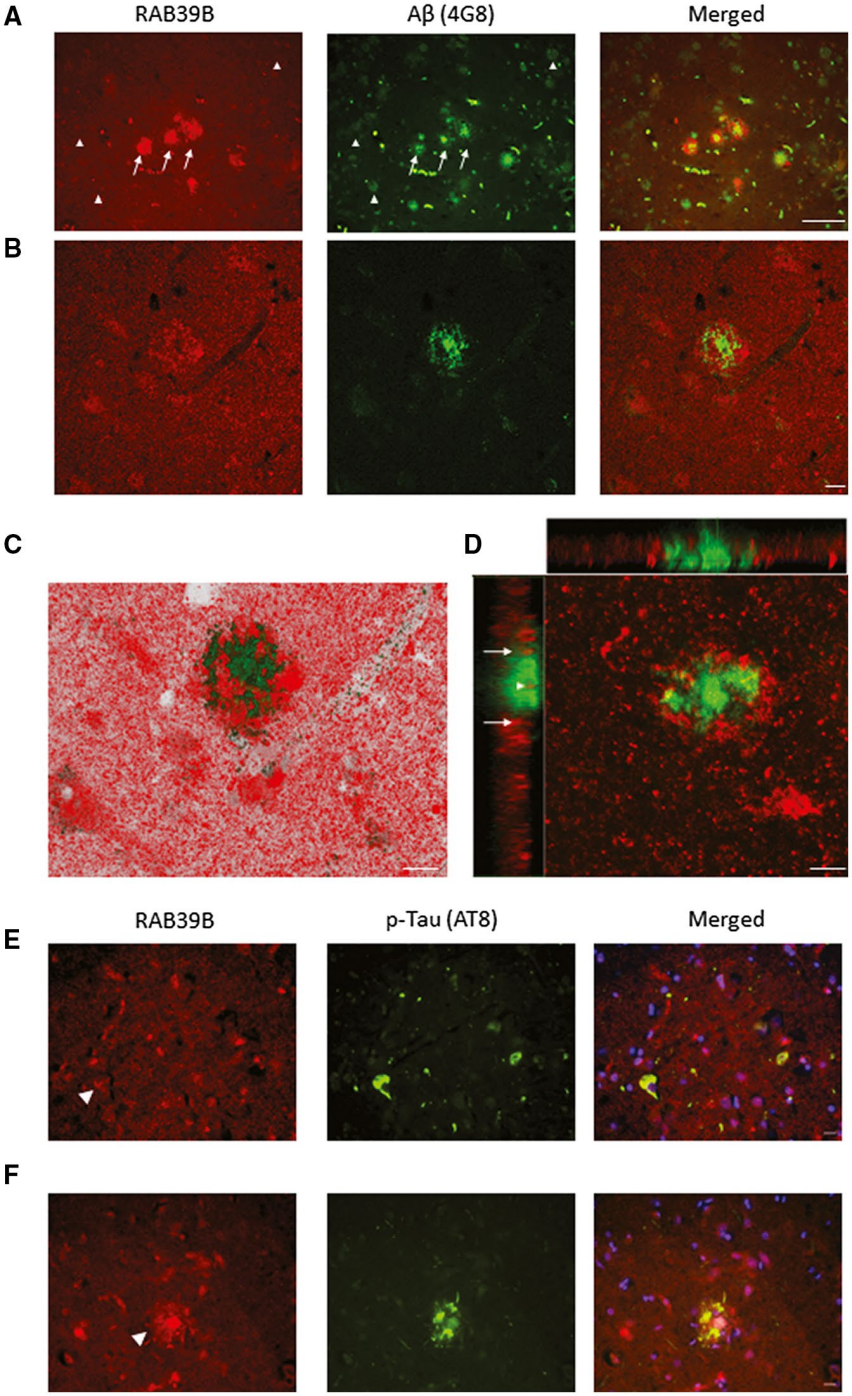
Co‐aggregation of RAB39B in Aβ plaques. Representative images of frontal cortex tissue stained for RAB39B (red) and Aβ (green) in a case of AD (**A**‐**D**). **A**. Wide‐field 200x fluorescence micrographs demonstrated the frequent co‐localization of RAB39B within cortical dense core Aβ plaques (arrows) in contrast to diffuse Aβ deposits (arrow heads) which were RAB39B negative. Co‐aggregation of RAB39B within dense core plaques was confirmed via confocal micrograph images (**B**‐**D**). Specifically, Z‐stack analysis (**D**) determined RAB39B sequestration within the core of the plaque (arrowhead) in addition to the accumulation of RAB39B at the periphery of the plaque (arrows). **E** and **F**. 400x micrographs of frontal cortex tissue stained for RAB39B (red) and AT8 positive phosphorylated tau (p‐Tau; green) are presented. **E.** No overt accumulation of RAB39B was observed in neurofibrillary tangle bearing neurons (arrowhead). **F**. Co‐localization of AT8 p‐Tau within RAB39B positive plaques was observed, indicative of neuritic subtype. Slides were coverslipped with DAPI containing mounting media (blue; **E**‐**F**). Scale bar in **A** = 50 µm and in **B**‐**F** = 10 µm

Numerous RAB39B positive Aβ plaques were revealed within examined cortical regions (Figure 2). The integration of RAB39B was evident in focal dense cored plaques with the accumulation of RAB39B evident at the periphery of the aggregates as well as within the center of some plaques (Figure 2A). More diffuse Aβ depositions (eg, fleecy plaques) did not show overt RAB39B reactivity (Figure 2A). Confocal imaging further supported the co‐aggregation of RAB39B with Aβ in focal plaques and supported the peripheral accumulation (Figure 2B,C) as well as the sequestration of RAB39B within the Aβ rich core as determined via Z‐stack visualization n (Figure 2D). RAB39B positive plaques were observed in all cases inspected independent of disease (n = 3 for Con, AD, DLB, see, Table S1 for specific cases used and Figure S2 for supportive images).

In contrast, to the accumulation of RAB39B within plaques, there was no modification of RAB39B distribution within AT8 positive NFT bearing neurons or neuritic threads (Figure 2E). However, extracellular plaque‐like structures positive for both AT8 reactive phospho‐tau and RAB39B were observed, consistent with the co‐aggregation of RAB39B within neuritic plaques (Figure 2F).

**Figure 3:**
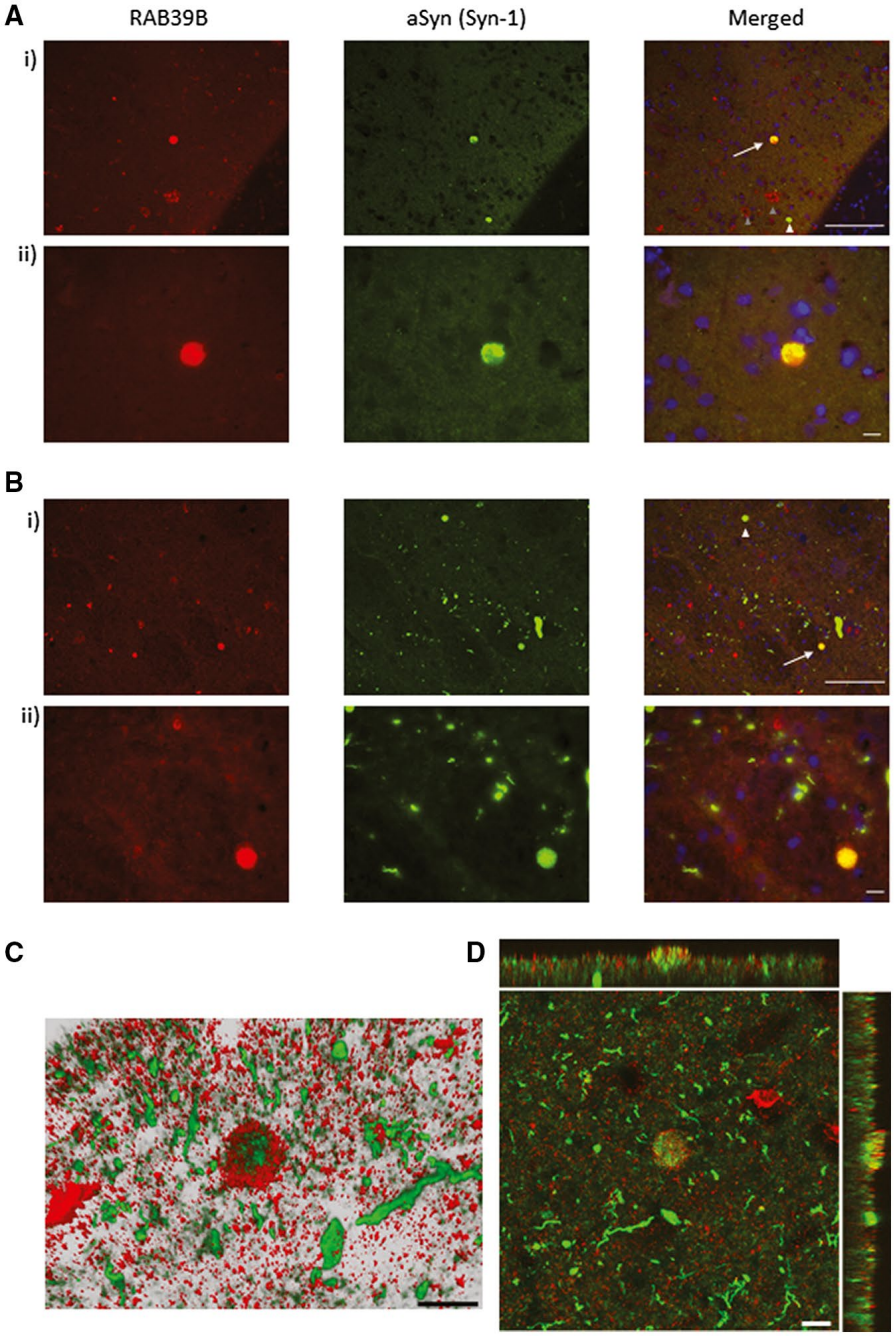
Co‐aggregation of RAB39B in aSyn LBs. Representative images of temporal cortex (**A**) and amygdala (**B**) stained for RAB39B (red) and aSyn (Syn‐1, green) with nuclei label with DAPI (blue) in a DLB case at 200x (i) and 400x magnification (ii). Wide‐field fluorescence micrographs demonstrated RAB39B positive Lewy bodies (LBs; arrows in i) and RAB39B negative LBs (white arrowheads in **A** i). Note RAB39B positive Aβ plaques are also visible in the cortex (gray arrowheads in **A** i). Co‐aggregation within LBs was confirmed via confocal imaging (**C** and **D**). Three dimensional reconstruction (**C**) and Z‐stack analysis (**D**) demonstrate selective RAB39B sequestration within LBs as oppose to Lewy neurites. Scale bar in **A** i + **B** i = 100 µm and in **A** ii + **B** ii and **C** and **D** = 10 µm

In DLB cases, the co‐localization of RAB39B with selective cortical and subcortical LBs was observed (Figure 3), while no RAB39B and aSyn co‐localization was seen in Lewy neurites (Figure 3A,B i,ii). Confocal imaging with 3D reconstruction (Figure 3C) and Z‐stack analysis (Figure 3D) further confirmed the co‐aggregation of RAB39B with aSyn within LBs. RAB39B/Syn‐1 structures were absent in any of the examined Con or AD cases (n = 3 for each neurodegenerative group, for an examples of physiological expression pattern see, Figure S2B ii).

### Total and subcellular distribution of RAB39B

**Figure 4:**
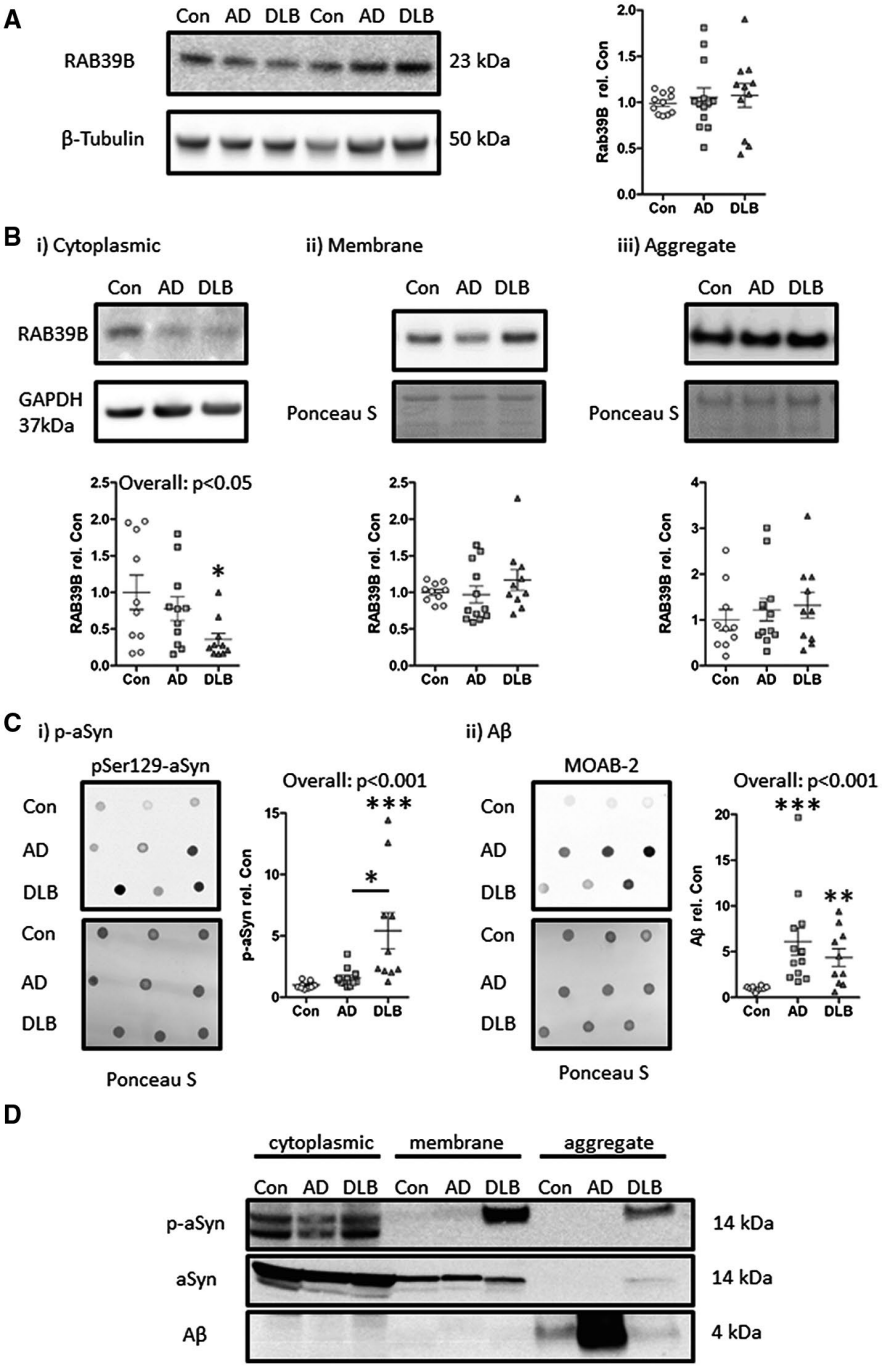
Altered subcellular distribution of RAB39B in DLB cases alongside elevated aSyn and Aβ pathology. Immunoblot quantification within crude whole tissue lysate (**A**) found for no overt change in absolute levels of RAB39B between control (Con), Alzheimer’s disease (AD) and dementia with Lewy bodies (DLB) cases. However, when examined in subcellular fractions (**B**), a significant reduction in cytoplasmic RAB39B was observed in DLB cases (i), despite comparable levels of RAB39B within the membrane (ii) and aggregate (iii) fractions. Loading controls of β‐tubulin, GAPDH and total protein via Ponceau S stain are shown. **C**. Quantification of aSyn pathology as per phospho‐serine 129 (pSer 129) aSyn antibody (p‐aSyn) incubated dot‐blot (i) and of Aβ via MOAB‐2 incubated dot‐blot (ii), demonstrated enhanced levels of p‐aSyn and Aβ in crude lysates of DLB cases compared to Con cases in contrast to AD cases where only Aβ was elevated. **D**. Visualization of aSyn and Aβ within cytoplasmic, membrane and aggregation fractions demonstrated the detection of p‐aSyn within the membrane and aggregation fraction only in the DLB case. Additionally, Aβ was largely confined to the aggregation fraction but was evident in all cases probed. Total aSyn is also shown for comparison. For western blots, all samples were run at 2 µg/lane except for the aggregated fraction which was run at 5 µL/lane. For dot‐blots, samples were dotted at 10 µL/dot. All data provided as scatter plots, with values expressed relative to mean con values ± SEM. *P < 0.05, **P < 0.01 and ***P < 0.001

Next, we assessed the total levels of RAB39B in crude tissue lysates of the temporal cortex. No overt alteration in RAB39B levels was observed (Figure 4A). However, fractionation of whole tissue lysate revealed a significant decline in cytoplasmic RAB39B levels, when adjusted for protein loading as per GAPDH, in DLB cases compared to controls (*P* < 0.05, Figure 4B i) but not in AD cases. Corresponding levels of RAB39B within membrane and aggregate fractions, adjusted for protein loading via Ponceau S total protein stain, were not statistically altered (Figure 4B ii,iii). No correlation with age was observed with RAB39B in either whole tissue lysates or any of the resulting fractions. However, a modest inverse relation with PMI and total RAB39B expression within the crude tissue lysate (r = 0.38, *P* < 0.05) was found, but not in cytoplasmic, membrane or aggregate fractions (*P *> 0.05).

Consistent with an association of RAB39B dysfunction with Aβ and aSyn pathology in DLB cases, the levels of p‐aSyn as determined via pSer129‐aSyn antibody (*P* < 0.001; Figure 4C i) and Aβ via the non‐APP cross reacting Aβ antibody MOAB‐2 (*P* < 0.01; Figure 4C ii), were increased in DLB cases compared to controls, within crude temporal cortex lysates. The elevation of both p‐aSyn and Aβ is in agreement with previous studies of cerebral amyloidosis in DLBs (30) and was in contrast, to AD cases where only Aβ (*P* < 0.001; Figure 4C ii) was raised. Linear correlation analysis failed to determine any significant correlation between p‐aSyn or Aβ and any measure of RAB39B however co‐localization of RAB39B with aSyn and Aβ pathology within the aggregated fraction, as well as with p‐aSyn in the membrane fraction, was established by western blot (Figure 4D).

## Discussion

Our data show that RAB39B expression is foremost altered in DLB, such that a loss of cortical RAB39B immunoreactivity was observed alongside a reduction in the levels of the protein in the cytoplasm as well as the co‐aggregation of RAB39B in LBs. In addition, we found widespread inclusion of RAB39B within dense core Aβ plaques.

In DLB, the decline in the cortical area positive for RAB39B together with its cytoplasmic reduction in the absence of overt changes in the total level of RAB39B argues for an altered distribution of the vesicular trafficking protein. This is in contrast, to the majority of LBD cases associated with RAB39B mutations and indeed the recent study of idiopathic PD (11) where an overall reduction in the expression of this protein was foremost evident (5, 16, 28, 35).

However, the PD‐linked G192R RAB39B mutation largely alters the cellular distribution without a reduction in total levels (19). Therefore, despite the absence of an overall decline, such redistribution of RAB39B from the cytoplasm is unlikely to happen without detriment and is consistent with other changes in localization that we recently reported (31). In the cytoplasm, RAB proteins are in an inactive state, either in reserve or in the process of cycling from one membrane to another (25, 33). Thus, our data suggest a reduction in the inactive pool of the protein and thus an increase of the available RAB39B that has been recruited to enact its associated trafficking functions. Consequently, impacted cells can be anticipated to carry a diminished maximal capacity for RAB39B mediated trafficking and, therefore, associated functions such as synaptic maintenance (13, 22) and aSyn homeostasis (14, 35) may go unmet when placed under high demand. Given the role of RAB39B in perturbing aSyn aggregation (14) the altered subcellular distribution may be compensatory. Alternatively or indeed additionally, the loss of RAB39B may reflect the trapping of the protein into the nonfunctional space of protein aggregates.

Nevertheless, we failed to find a significant elevation of membrane‐ or aggregate‐associated RAB39B reciprocal to the loss of cytoplasmic RAB39B in DLB cases. One possible explanation for this, is that the combinatorial effect of more modest alterations in membrane association and sequestration into aggregates leads to the significant decrease observed in cytoplasmic levels. Indeed, in the context of DLB cases, we see an increases in both the amount of RAB39B in the membrane fraction and aggregation fraction, though neither change reaches statistical significance. Of course, it must also be considered that in the presence of aSyn and Aβ pathology an increase in RAB39B turnover or a reduction in RAB39B expression may occur, although such an explanation would be in contrast to the unaltered levels of total RAB39B expression established from measurements here within crude lysates.

To the best of our knowledge, this is the first report of RAB39B and aSyn co‐aggregation, although a number of other RAB proteins have been detected within LBs via proteomic approaches (17, 36) and via immunocytochemistry in our own work in cell models (14). The factors that dictate the presence or absence of RAB39B in these LBs are unclear, but may reflect the occurrence of LBs within a specific neuronal subtype, the maturation state of the aggregate or the inopportune random trapping of RAB39B vesicles which may have been attempting to limit aSyn aggregation. Given recent ultrastructure analysis of LBs which found heterogeneous lipid membrane compositions within the core of these aggregates (27), the inclusion of RAB39B‐associated membranes within a subset of LBs would appear highly plausible.

Regardless, impairments to the controls of aSyn aggregation, caused by the sequestration of RAB39B when already in the presence of lewy pathology, may in turn establish a positive feedback loop facilitating a greater degree of local intracellular aSyn deposition.

The mode of involvement of RAB39B in the pathology of idiopathic LBDs, as supported by our data, is somewhat inconsistent with a recent study reporting a ~70% reduction in total levels of RAB39B within the pre‐frontal cortex of idiopathic PD cases (11). Combined, the contradictory data may suggest a divergence of mechanisms between LBD subtypes, a reduction of RAB39B levels in PD and a redistribution of RAB39B in DLB. Notwithstanding the potential for regional differences in the mechanisms which drive the dysfunction of RAB39B, such a determination of a decline in the total levels of RAB39B may be dependent on the existence of age‐related neuropathology within the control group. Given that we observed the integration of RAB39B into Aβ plaques in all cases examined, it is feasible that the extent of age‐associated cerebral Aβ pathology may influence the outcome of comparisons between disease and control groups. In the absence of published data relating to such age‐related pathology within the recent study of RAB39B in idiopathic PD further analysis of the issue is limited (11).

Although, proteomic analysis has previously identified RAB39B as a component of Aβ plaques in high‐pathological controls, AD cases and those of AD mouse models (7, 38), here such findings are confirmed via immunohistochemistry. RAB39B positive plaques were observed with high frequency throughout the investigated cortical regions. Focal plaques, particularly but not exclusively phospho‐tau positive neuritic plaque subtypes were most robust in RAB39B reactivity. In contrast, diffuse Aβ aggregates, which lack an association with dystrophic neurites and cell bodies (8), were largely RAB39B negative. Thus, the difference in reactivity profiles between these plaque subtypes suggests that the inclusion of RAB39B containing structures occur as part of the trapping of cytoplasmic and membranous components of neuronal processes. Nevertheless, the sequestration of RAB39B within age associated aggregates raises the potential for RAB39B functions to decline as a consequence of aging, thus predisposing the brain for protein aggregation and neurodegenerative onset.

It is possible that both aSyn and Aβ aggregation impact upon RAB39B homeostasis and only when combined is the redistribution of RAB39B widely apparent. Consistent with this hypothesis, we here observed that both aSyn and Aβ pathology was elevated in our DLB cohort, alongside the loss of RAB39B from the cytoplasmic fraction. However in light of the lack of a direct linear relationship between either aSyn or Aβ pathology and any measure of RAB39B, a more detailed investigation of pathological species (eg, soluble vs. aggregated) may be required or equally the use of mixed modeling to account for the interaction of factors, such as LB and plaque load but also age. Such future work would necessitate the use of a considerably larger sized cohort, than has been employed in this initial study.

Nevertheless, it is intriguing to note that in addition to the association of RAB39B with aSyn and Aβ, as observed here and elsewhere (14, 35), the protein has also been implicated in huntingtin protein homeostasis (39) and has a clear functional dependency on C9orf72, which is associated with ALS and FTD (9, 26, 37). Thus, dysfunctions in RAB39B trafficking as a consequence of age‐associated protein aggregation may well contribute to an array of neurodegenerative diseases. As such further investigation of the altered nature of RAB39B within the broader scheme of neurodegeneration or indeed physiological aging is warranted as is the consideration of RAB39B and or its associated functions as targets for future therapeutic treatment.

In conclusion, the loss of RAB39B as a consequence of age‐associated cerebral Aβ pathology and LB formation likely impedes RAB39B functions associated with aSyn homeostasis and synaptic integrity, in turn, contributing to protein aggregation and neuronal dysfunction. The prominent accumulation in RAB39B in Aβ plaques may specifically facilitate aSyn LB deposition and thus, may be contributory to the age‐associated vulnerability of LBD onset.

## Conflict of Interests

The authors declare that they have no competing interests.

## Author Contributions

DJK, OB, SA and ML conducted the experiments. DJK, FG and TFO designed the study, DJK, FG, JA and TFO wrote the manuscript. All authors read and approved the final manuscript.

## Supporting information

Fig S1
**Figure S1.** **Regional staining of RAB39B.** Representative micrographs stained for RAB39B and captured at 100X magnification from a selected control case. Images are shown of the prefrontal (pFc), frontal (Fc), Cingulate (Cc), Insular (Ic), Motor (Mc), Entorhinal (Ec), Temporal (Tc), Parietal (Pc) and Occipital cortex (Oc) as well as of the Amygdala (Amyg) and frontal white matter (WM). Scale bar = 50 µm.

Fig S2
**Figure S2.** **Supporting fluorescent micrographs of RAB39B co‐aggregation.** **A.** a selection of cortical Aβ plaques also positive for RAB39B immunoreactivity captured from slides prepared from control (i), Alzheimer’s disease (AD; ii) and dementia with lewy bodies (DLB; iii) cases. **B**. Cortical tissue stained for aSyn and RAB39B and nuclei co‐labeled via a DAPI stain, demonstrated a pattern of RAB39B staining consistent in morphology to Aβ plaques as seen in a DLB case (i). This was in contrast to non‐pathological, physiological expression pattern for both RAB39B and aSyn as captured from cortical sections of a control case (ii). Scales in **A** i, **B** = 10 µm and in **A** ii,iii = 50 µm.

Table S1
**Table S1.** **Human cases used in this study.** Diagnosis (Diag), age (in years), sex, post mortem interval (PMI, in hrs) and neuropathological assessment scores for neurofibrillary tangle (NFT) Braak stage, Thal phase, Consortium to Establish a Registry for Alzheimer’s Disease (CERAD), the National Institute of Aging––Alzheimer’s Association (NIA‐AA) criteria, Lewy body (LB) Braak stage and McKeith criteria are provided. For McKeith criteria, absence of Lewy pathology (No LB), Amygdala predominate, Limbic predominate and Neocortical predominate are indicated. The use of each case in tissue microarray (TMA), multi‐channel fluorescence histochemistry (Histo) and/or immuno‐blots (IB) are also listed. NA= not available.

## Data Availability

The datasets used and/or analyzed during the current study are available from the corresponding author on reasonable request.
