# Supplementary material for: RAB39B is redistributed in dementia with Lewy bodies and is sequestered within aβ plaques and Lewy bodies
Source: Brain Pathol. 2020 Aug 25;31(1):120–32. doi: 10.1111/bpa.12890 (PMC8018064; doi:10.1111/bpa.12890)
Supplement: Supplementary file 3 — Table S1 Table S1. Human cases used in this study. Diagnosis (Diag), age (in years), sex, post mortem interval (PMI, in hrs) and neuropathological assessment scores for neurofibrillary tangle (NFT) Braak stage, Thal phase, Consortium to Establish a Registry for Alzheimer’s Disease (CERAD), the National Institute of Aging––Alzheimer’s Association (NIA‐AA) criteria, Lewy body (LB) Braak stage and McKeith criteria are provided. For McKeith criteria, absence of Lewy pathology (No LB), Amygdala predominate, Limbic predominate and Neocortical predominate are indicated. The use of each case in tissue microarray (TMA), multi‐channel fluorescence histochemistry (Histo) and/or immuno‐blots (IB) are also listed. NA= not available. [file BPA-31-120-s003.rtf]

Case	Diag	age	sex	PMI	NFT Braak	Thal	CERAD	NIA-AA	LB Braak	McKeith	Usage	
1	Con	84	M	45	II	3	0	1	0	No LB	TMA+IB	
2	Con	81	F	75	II	4	0	1	0	No LB	TMA+Histo+IB	
3	Con	52	M	102	0	0	0	0	0	No LB	TMA+WB	
4	Con	80	F	31	III	2	0	1	0	No LB	TMA+Histo+IB	
5	Con	64	M	93	I	0	0	0	0	No LB	TMA+IB	
6	Con	99	F	5	II	0	0	0	0	No LB	TMA+IB	
7	Con	92	M	50	III	1	0	1	0	No LB	TMA+IB	
8	Con	73	M	25	0	0	0	0	0	No LB	TMA+IB	
9	Con	78	F	34	0	1	0	1	0	No LB	TMA+IB	
10	Con	70	F	72	0	1	0	1	2	No LB	TMA+Histo+IB	
11	Con	88	F	22	III	0	0	0	0	No LB	TMA	
12	Con	80	M	25	II	1	0	1	0	No LB	TMA	
13	AD	93	M	22	VI	5	3	3	0	Amygdala 	IB	
14	AD	86	M	9	VI	5	3	3	0	No LB	IB	
15	AD	89	F	11	VI	5	3	3	0	No LB	TMA+IB	
16	AD	80	M	39	VI	4	3	3	0	No LB	TMA+IB	
17	AD	81	M	41	VI	5	3	3	0	Amygdala 	TMA+IB	
18	AD	92	F	74	VI	5	3	3	0	No LB	TMA+Histo+IB	
19	AD	89	M	61	VI	5	3	3	0	No LB	TMA+Histo+IB	
20	AD	80	M	24	VI	5	3	3	0	No LB	TMA+IB	
21	AD	86	F	5	VI	5	3	3	0	No LB	TMA+IB	
22	AD	86	F	47	VI	5	3	3	0	Amygdala 	TMA+IB	
23	AD	78	F	37	VI	5	3	3	0	Amygdala 	TMA+IB	
24	AD	77	F	63	VI	5	3	3	0	No LB	TMA+Histo+IB	
25	DLB	84	M	72	II	3	0	1	6	Neocortical	TMA+Histo+IB	
26	DLB	74	M	60	II	5	0	1	5	Neocortical	TMA+Histo+IB	
27	DLB	73	M	47	III	1	0	0	6	Neocortical	Histo+IB	
28	DLB	71	M	22	III	4	2	2	6	Neocortical	TMA+IB	
29	DLB	81	M	26	III	3	2	2	6	Neocortical	TMA+IB	
30	DLB	78	M	8	III	4	2	2	6	Neocortical	TMA+IB	
31	DLB	73	F	99	III	4	0	1	6	Neocortical	IB	
32	DLB	81	M	24	IV	N.A	1	1	6	Neocortical	TMA+IB	
33	DLB	91	F	84	V	N.A	3	N.A	N.A	Limbic	TMA+IB	
34	DLB	75	F	63	VI	N.A	3	N.A	N.A	Neocortical	TMA+IB	

Supplemental Table 1. Human cases used in this study. Diagnosis (Diag), age (in years), sex, post mortem interval (PMI, in hrs) and neuropathological assessment scores for neurofibrillary tangle (NFT) Braak stage, Thal phase, Consortium to Establish a Registry for Alzheimer's Disease (CERAD), the National Institute of Ageing – Alzheimer's Association (NIA-AA) criteria, Lewy body (LB) Braak stage and McKeith criteria are provided. For McKeith criteria, absence of Lewy pathology (No LB), Amygdala predominate, Limbic predominate and Neocortical predominate are indicated. The use of each case in tissue microarray (TMA), multi-channel fluorescence histochemistry (Histo) and/or immuno-blots (IB) is also listed. N.A= not available.
	
